# Immobilization of Glucose Oxidase in Alginate-Chitosan Microcapsules

**DOI:** 10.3390/ijms12053042

**Published:** 2011-05-11

**Authors:** Xia Wang, Ke-Xue Zhu, Hui-Ming Zhou

**Affiliations:** 1 State Key Laboratory of Food Science and Technology, Jiangnan University, 1800 Lihu Avenue, Wuxi 214122, Jiangsu, China; E-Mails: hawangxia@yahoo.cn (X.W.); kxzhu@jiangnan.edu.cn (K.-X.Z.); 2 School of Food Science and Technology, Jiangnan University, 1800 Lihu Avenue, Wuxi 214122, Jiangsu, China

**Keywords:** glucose oxidase, alginate, chitosan, microcapsules, encapsulation efficiency

## Abstract

In order to improve its stability and catalytic rate in flour, the immobilization of glucose oxidase (GOX) was investigated in this work. The enzyme was encapsulated in calcium alginate-chitosan microspheres (CACM) using an emulsification-internal gelation-GOX adsorption-chitosan coating method. The interaction between alginate and chitosan was confirmed by infrared spectroscopy (IR). The resultant CACM in wet state, whose morphology was investigated by scanning electron microscopy (SEM), was spherical with a mean diameter of about 26 μm. The GOX load, encapsulation efficiency and activity of the CACM-GOX were influenced by concentration of chitosan, encapsulation time and encapsulation pH. The highest total enzymatic activity and encapsulation efficiency was achieved when the pH of the adsorption medium was near the isoelectric point (p*I*) of GOX, approximately pH 4.0. In addition, the molecular weight of chitosan also evidently influenced the encapsulation efficiency. Storage stabilities of GOX samples were investigated continuously over two months and the retained activity of CACM-GOX was 70.4%, markedly higher than the 7.5% of free enzyme. The results reveal the great potential of CACM-GOX as a flour improver.

## 1. Introduction

Applying enzymes instead of chemical oxidants is a very interesting option to improve the bread-making performance of dough since they are considered as natural and non-toxic food components. Currently, the baking industry is deeply involved in research on alternatives to potassium bromate due to its potential hazards. Glucose oxidase (GOX) (EC 1.1.3.4) is the preferred enzyme alternative to chemical oxidizing agents for bread improvement and has been cited for commercial use. It is a dimeric protein with a molecular weight of 160 kDa, containing one tightly but noncovalently bound flavin adenine dinucleotide (FAD) per monomer as cofactor and glycosylated with a carbohydrate content of 16% (w/w). In the presence of oxygen, GOX catalyzes the oxidation of β-d-glucose to β-d-gluconolactone and generates hydrogen peroxide (H_2_O_2_). The gluconolactone is then slowly hydrolyzed to gluconic acid via a nonenzymatic mechanism. The generated H_2_O_2_ is able to oxidize thiol groups into disulfide bonds and catalyze the water-soluble pentosans in the dough to gel in the presence of peroxidase, which explains its improving effect on wheat flour [1–7].

There are still some deficiencies of GOX despite its good effects on flour, and its encapsulation and immobilization has being studied due to its high oxidization rate and low stability in flour. As a fast-acting oxidant, GOX reduces the elasticity of dough immediately after mixing because of the abundant H_2_O_2_ generated during that period, which accounts for the dry and strong dough as well as bread with low volume and poor crumb [8–10]. Meanwhile, according to Rakotozafy *et al.* [11], 25% of the GOX activity was lost in the first 5 min of mixing with an additional loss of 20% observed after 20 min based on the assumption of a physicochemical denaturation in the dough environment.

The basic idea behind enzyme immobilization is to entrap the protein in a semi-permeable support material that prevents the enzyme from leaving while allowing substrates, products and co-factors to pass through. The catalytic rate of the enzyme inside the microcapsules could be adjusted by the thickness of its wall material. Compared with free enzymes in solution, the immobilized ones are more robust and resistant to environmental changes. The immobilized GOX in the microspheres can not only prevent enzyme from denaturing but can also delay the generation of H_2_O_2_ in dough.

Alginate is by far the most widely used polymer in immobilization and micro-encapsulation technologies [12,13]. Alginate is a seaweed extract composed of chains of alternating α-l-guluronic acid (G) and β-d-mannuronic acid (M) residues. Alginate supports are usually made by cross-linking the carboxyl group of the α-l-guluronic acid with a solution of a cationic cross linker such as calcium chloride. In the experiment, an internal gelation method was selected [14,15]. Insoluble calcium carbonate was added into an alginate solution and the mixture was added into vegetable oil containing surfactant and stirred at high speed resulting in formation of a W/O emulsion. The gelation reaction was initiated by dropping the pH of the W/O emulsion to release the calcium ions. Calcium alginate (CaAlg) gel beads were formed immediately upon contact between the alginate and calcium ions by forming an ionic complex. GOX penetrates to the interior of CaAlg bead and binds to the surface of the alginate chains through ionic and hydrogen bonds [16–18].

Chitosan is another natural polymer that has gained tremendous interests in immobilization technology. Chitosan is used either as a means of coating alginate beads in order to alter the diffusion rate of the GOX [19] or as an additive to prevent the enzyme from escape [12,20].

The purpose of the present work was to encapsulate GOX into CACM with high encapsulation efficiency and residual activity. Immobilization conditions including the concentration and molecular weight of chitosan, encapsulation pH and absorption time were optimized. Thermal and storage stabilities of CACM-GOX compared with that of free enzyme were also studied in the paper.

## 2. Materials and Methods

### 2.1. Materials

All chemicals and reagents used in this work were food-grade or reagent-grade. Glucose oxidase (Gluzyme Mono) containing 10,000 glucose oxidase units/g was kindly supplied by Novo Nordisk (Shanghai, China). Alginate (sodium salt, viscosity of 1.5% solution at 30 °C is 2100 cps) was purchased from Sinopharm Chemistry Reagent (Co Ltd., Shanghai, China). Chitosans with different molecular weights and 91% deacetylation degree were purchased from Yuhuan Ocean Biochemistry (Co Ltd., Zhejiang, China).

### 2.2. Microsphere Preparation

A modified emulsification-internal gelatin method [14,17,21] was applied to prepare CaAlg gel beads. 0.08 g micro-crystalline calcium carbonate was added into 20 mL sodium alginate solution of 1.5% (w/v) and placed in an ultrasonic bath for 5 min using an Ultrasonic Microwave Assisted Extraction (CW-2000, Xintuo, Shanghai, China). The suspension was dispersed into 40 mL soybean oil containing Span 80 of 2% (v/v) by stirring at 10,000 rpm. After emulsification for 2 min, 30 mL soybean oil containing Span 80 and 0.2 mL of glacial acetic acid were added and stirring continued for 10 min. Then 250 mL distilled and deionized water was added into the above emulsion, stirring continued for 30 min at a speed of 400 rpm. The calcium alginate (CaAlg) gel beads were washed twice with 200 mL Tween 80 solution of 2% (v/v) and then several times by 200 mL deionized water to remove any traces of oil until no residual oil was observed by optical microscope.

One milliliter GOX of 1 mg/mL in a 0.2 M buffer solution of pH 4 (sodium acetic-acetic acid) was added into 2 mL CaAlg gel beads (dry weight of 7.7 mg/mL). The adsorption experiments were carried out for 60 min at 4 °C, followed by incubating the GOX CaAlg gel beads in 1 mL 1% (w/v) chitosan acetic acid solution (1%, v/v) for 10 min at 4 °C, shaking quickly to ensure the reaction uniform. The CACM beads (0.74 mL, dry weight of 24.9 mg) were washed twice with 10 mL deionized water and blended with 0.26 mL 0.2 M acetic buffer solution of pH 6 for activity assay.

### 2.3. Activity Assay for Free and Encapsulated GOX

The activities of free GOX and CACM-GOX were measured by the DNSA method [21,22]. The enzyme assay mixture consisted of 1 mL β-d-glucose solution (1 mg/mL) and 0.1 mL free enzyme or appropriate volume of other enzyme preparation followed by adding acetic buffer (pH 6.0) to 4 mL. The mixture was incubated at 30 °C for 30 min under a vibrating state in a rocking bed. The reaction was stopped by holding the tube in boiling water. To measure residual sugar, 2 mL DNSA reagent was added into the above tube and the mixture was boiled for 5 min followed by cooling to room temperature and diluted to 25 mL. The latter was quantified by measuring absorbance at 520 nm using a spectrophotometer (Spectronic 2000: Milton Roy, USA). A unit of glucose oxidase activity is defined as the amount of enzyme which oxidizes 1.0 μmol of β-d-glucose to gluconic and hydrogen peroxide per minute at 30 °C.

### 2.4. Characterization of CACM

Microsphere features such as shape and existence of aggregates were examined after isolation by an optical microscope Olympus CKX41 equipped with a digital camera (Olympus DP12, Japan). CACM was stained by neutral fuchsin. Scanning electron microscopy (Quanta-200, FEI, Holland) was utilized to observe the morphology of coated microspheres. The particle size and size distribution of the CaAlg gel beads and the CACM were determined by a particle size analyzer (Mastersizer 2000, Malvern, Worcestershire, UK).

Infrared (IR) spectra of alginate, chitosan, and CACM were recorded with a Fourier transform infrared spectrometer (270-30, Hitachi, Wuxi, China). Freeze-dried samples were scanned from 600 to 4000 cm^−1^ at a resolution of 4 cm^−1^.

### 2.5. Optimization of Immobilization Conditions

#### 2.5.1. Encapsulation pH

To investigate optimum encapsulation pH and the total activity of CACM-GOX, 2 mL CaAlg gel beads (dry weight of 7.7 mg/mL) were added into 1 mL GOX of 1 mg/mL prepared in 0.2 M acetate buffer at pH 3.0, 3.5, 4.0, 4.5, 5.0 and 0.2 M phosphate buffer at pH 6.0 and 7.0. Encapsulation mixture was kept at 4 °C for 80 min. The experiment revealed that GOX was stable under pH 4.5 and 5.0 and no deactivation or precipitation was observed. The encapsulation efficiency was calculated in terms of the change of GOX activity in the supernatant before and after incubation of CaAlg gel beads in enzyme solutions.

(1) $$\text{Encapsulation}\;\text{efficiency}\;(\%)=\frac{\text{Total}\;\text{GOX}\;\text{activity}\;\text{added-GOX}\;\text{activity}\;\text{in}\;\text{supernatant}}{\text{Total}\;\text{GOX}\;\text{activity}\;\text{added}}\times 100\%$$

#### 2.5.2. Chitosan Molecular Weight

One percent (w/v) chitosan solutions were prepared by dissolving chitosans, which had the same degree of deacetylation (91%) but different molecular weight of 20,000 (LMW), 100,000 (MMWa), 200,000 (MMWb) and 450,000 (HMW), into 1% acetic acid solution. The GOX adsorption experiments were carried out as above followed by incubating the GOX CaAlg gel beads in each chitosan solution for 10 min at 4 °C.

#### 2.5.3. Encapsulation Time and Chitosan Concentration

Chitosan acetic acid solutions of 0.5%, 1% and 2% with a predetermined molecular weight were prepared. 1 mL GOX solution was added into 2 mL CaAlg gel beads. Encapsulations were carried out for 10, 20, 40, 60 and 80 min separately, followed by incubation in each chitosan acetic acid solution (pH 4.0) for 10 min. The amounts of GOX loaded and activities of CACM-GOX were determined based on dry weight of the particles. The amount of GOX loaded was calculated by the following equation. Protein values were determined by the method of Lowry.

(2) $$\text{The}\;\text{amount}\;\text{of}\;\text{GOX}\;\text{loaded}\;(\text{mg}/\text{g})=\frac{\text{The}\;\text{protein}\;\text{weight}\;\text{in}\;\text{CACM}(\text{mg})}{\text{Total}\;\text{weight}\;\text{of}\;\text{dry}\;\text{CACM}\;\text{particles}(\text{g})}$$

### 2.6. Thermal and Storage Stabilities of Free GOX and CACM-GOX

Free GOX solution containing 1 mg/mL enzyme protein in 0.2 M buffer (pH 6.0) and CACM-GOX (dry weight of 33.65 mg/mL, pH 6.0) with an activity of 8.86 U/mL were kept at 4 °C and 25 °C, and the residual activities of these samples were measured periodically for 60 days and 60 hours, respectively.

### 2.7. Statistical Analysis

Each experiment was repeated three times. The results were expressed as means ± S.D. and analyzed by SAS (a statistical computer program; version 8) analysis of variance method.

## 3. Results and Discussion

### 3.1. Characterization of CACM

**Figure 1.**
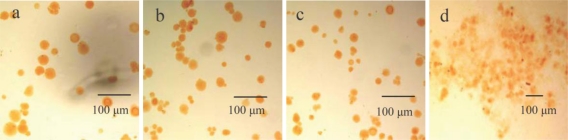
Optical micrographs of wet CaAlg beads (**a**), calcium alginate-chitosan microspheres (CACM) coated with 0.5% chitosan (**b**), CACM coated with 1.0% chitosan (**c**) and CACM coated with 2.0% chitosan (**d**).

The morphology of microspheres was determined in terms of their final shape and granulometry. Wet CaAlg beads presented as spherical by optical micrographs (Figure 1a), CACM coated with chitosans of 0.5% and 1.0% showed similar spherical shape (Figure 1b,c), while the existence of clustered alginate microspheres surrounded by chitosan coacervates was observed by microscope during coating CaAlg beads with chitosan of 2.0% (Figure 1d).

**Figure 2.**
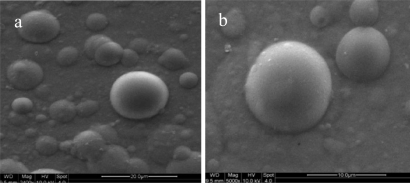
Scanning electron micrographs of calcium alginate-chitosan microspheres**-**glucose oxidase (CACM-GOX).

The micrographs of calcium-alginate microspheres (MMWb chitosans of 1.0%) of about 30 μm and approximate to regular spherical shape with a smooth external surface are shown in Figure 2a,b, being different from what was observed in Figure 1. Long chains of chitosan connecting two or more CaAlg beads together were also found in the picture, which accounted for the flocculent aggregation of CACM in Figure 1d. The surface of CACM became quite smooth due to water absorption and swelling caused by its abundant hydrogen bonding (Figure 2b).

**Figure 3.**
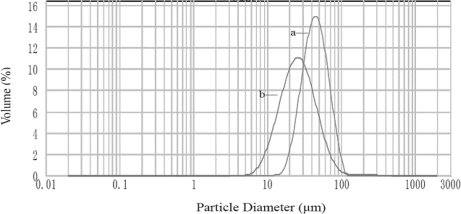
Particle size distribution of CaAlg beads (**a**) and CACM (**b**) particle diameters reported as volume-based in water.

The particle size and size distribution of a representative preparation of CaAlg beads and CACM are presented in Figure 3. Their volume-based mean diameters in the wet state were 44 μm and 26 μm, respectively, and their residual errors were 20.86% and 16.88%, respectively. The microspheres coated with chitosan were smaller than uncoated ones, due to the enwrapping and frapping effects of chitosan on microcapsules.

**Figure 4.**
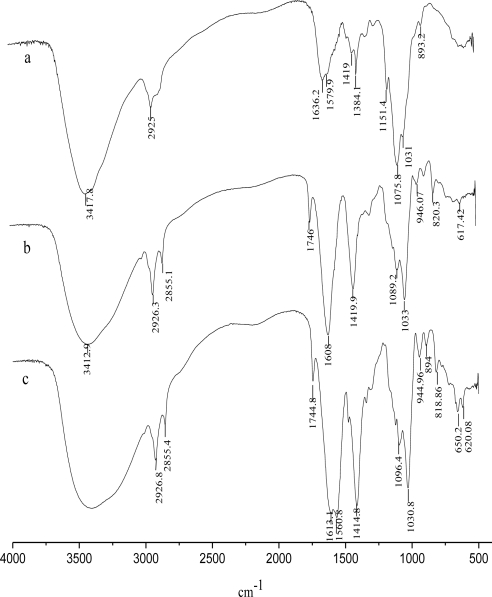
The infrared spectroscopy (IR) spectra of CaAlg beads (**a**), chitosan (**b**) and CACM (**c**).

In order to confirm alginate-chitosan interactions, samples were analyzed by IR spectroscopy. Figure 4 shows spectra of alginate (a), chitosan (b) and alginate-chitosan microspheres (CACM) (c). The IR spectrum of chitosan showed a band of C–H stretching at 2925 cm^−1^ and the absorption band of the N-H of non-acylated 2-aminoglucose primary amines bending vibration at 1570 cm^−1^. The peaks at 1419 and 1384 cm^−1^ belonged to the N-H stretching of the amide and ether bonds and N-H stretching (amide III band), respectively [23]. The bridge oxygen (C–O–C, cyclic ether) stretching bands at 1151, 1076, 1031, and 893 cm^−1^ were observed as well. The carbonyl (C=O) stretching of the secondary amide (amide I band) at 1655 cm^−1^ and the bending vibrations of the N–H (N-acetylated residues, amide II band) at 1558 cm^−1^ [23] were very weak because of a high degree of deacetylation. As a carboxyl salt, sodium alginate showed strong absorption bands at 1608 and 1420 cm^−1^ due to carboxyl anions (asymmetric and symmetric stretching vibrations).

For the alginate-chitosan beads, the band around 3500–3100 cm^−1^ became broader, which illustrated hydrogen bonding was enhanced [24]. Moreover, the N–H bending vibration of non-acylated 2-aminoglucose primary amines (band at 1570 cm^−1^) and asymmetric and symmetric –C–O stretching at 1636 and 1384 cm^−1^, respectively, disappeared, indicating that the –NH_2_^+^ of the chitosan had reacted with the –COO^−^ of the alginate. A new peak at 1561 cm^−1^ was assigned to the amide bond of CACM [18].

### 3.2. Optimization of the Encapsulation Conditions

#### 3.2.1. Effects of pH on GOX Encapsulation

**Figure 5.**
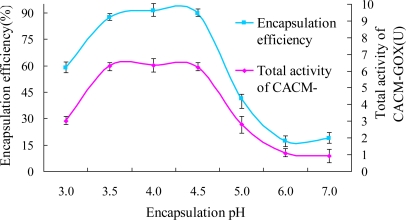
Effect of encapsulation pH on total activity of CACM-GOX and encapsulation efficiency.

To establish the optimum pH value for the encapsulation of GOX into CaAlg, the pH of encapsulation medium was changed between 3.0 and 7.0. As seen in Figure 5, the amount of GOX loaded was remarkably affected by the medium pH ranging from 3.0 to 7.0. The maximum GOX load occurred at pH 4.5. At pH values higher than 4.5 GOX load decreased rapidly with pH increasing up to 7.0. At pH 3 the GOX encapsulation efficiency was merely 58.6%, much lower than that of 91.1% at pH 4.0. These results are in good agreement with those reported by Ozyilmaz, Tukel and Alptekin [25], according to whom it may be due to electrostatic repulsive forces existing between the GOX molecules bound onto CaAlg and unbound free ones with the same charge. Another possible cause is that the p*K*a values of β-d-mannuronic acid (M) and α-l-guluronic acid (G) of alginate were 3.38 and 3.20, respectively. Therefore, both alginate and GOX were positively charged at pH 3, which was lower than the p*I* of alginate (pH 3.3), leading to low encapsulation efficiency due to electric repelling. A similar phenomenon occurs at pH values higher than 4.5 due to repulsive electrostatic interaction between negative charges. The total activity of CACM-GOX displayed the same trend with the immobilization efficiency, which suggested that GOX had good stability and low loss in the 80 min absorption process within the pH range of 3.0 to 7.0.

#### 3.2.2. Effect of Chitosan Molecular Weight on GOX Encapsulation

**Figure 6.**
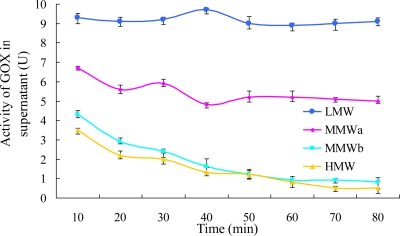
Effect of chitosan molecular weight on GOX encapsulation efficiency.

Four chitosan solutions with different molecular weights were selected to investigate the effects of molecular weight on the activity of GOX in the supernatant. The latter was calculated by measuring the difference between the total activity of GOX added in the preparation medium and the residual activity remaining in the aqueous supernatant suspension after the absorption process. As shown in Figure 6, the encapsulation efficiency decreased with decreasing molecular weight. The maximum encapsulation efficiency of 93.6% was achieved with the highest molecular weight (HMW) chitosan, while that of LMW was less than 10%. This could be attributed to the fact that chitosan with low molecular weight has a shorter molecular chain and smaller volume compared with GOX (160 kDa) and could diffuse deeply into the gel core [26], resulting in desorption of GOX from CaAlg beads. Whereas high molecular weight chitosan with a long chain could just coat on the surface of alginate beads [26] and form a more uniform lattice network enwrapping GOX in microcapsule, improving the encapsulation efficiency.

The encapsulation efficiency increased rapidly in the initial 20 min of absorption time and then slightly rose up to 80 min for the chitosans with high molecular weight. Chitosans of MMWb and HMW had similar variation law of encapsulation efficiency with high efficiencies of 93.1% and 91.1%, respectively, at 60 min. The egg-box structure formed by Ca^2+^ and alginate resulted in many channels, beneficial to entrance of GOX into CaAlg beads [18,27]. The viscosity of chitosan increased with increasing molecular weight, which caused an aberrant morphology, inhomogenous granularity and easy polymerization [25]. Therefore, chitosan MMWb was selected in latter experiments.

#### 3.2.3. Effects of Encapsulation Time and Chitosan Concentration on GOX Immobilization

The effects of chitosan concentration and encapsulation time on the encapsulation efficiency, the amount of GOX loaded and the total activity of CACM-GOX were evaluated. As seen in Table 1, GOX encapsulation efficiency was significantly (*p* < 0.01) affected by the concentration of chitosan. It increased as the reaction proceeded for up to 80 min and was at the greatest rate within the first 20 min for all the three concentrations and almost remained constant for periods longer than 60 min, which indicated that the enzyme absorption to the CaAlg beads happened mainly at the beginning followed by lower speed as the concentration of free enzyme decreased. The encapsulation efficiency was also influenced significantly by chitosan concentration (*p* < 0.05) and achieved the minimum in the presence of 0.5% chitosan. Through measuring the protein concentration in the supernatant of water before and after washing CACM-GOX, it was found that GOX could not be effectively mobilized in microcapsules and was prone to being washed out in the presence of low-concentration chitosan, leading to lower GOX load and activity of CACM-GOX.

The amount of GOX loaded and the total activity of CACM-GOX also increased gradually with extending encapsulation time due to more and more protein absorption onto CaAlg beads. They were also influenced by the concentration of chitosan as the total weight of CACM increased due to more chitosan coating on the surface of CaAlg beads with increasing concentration. The phenomenon that chitosan competed with GOX for absorption [17] did not happen to chitosans with high molecular weight.

**Figure 7.**
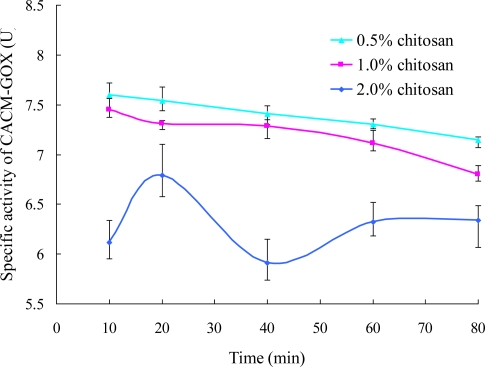
Effect of encapsulation time on specific GOX activity. The specific activity of CACM-GOX is the ratio of total activity of CACM-GOX to encapsulation efficiency.

The specific CACM-GOX activity, to measure the catalytic rate of the immobilized enzyme, was taken as the ratio of total CACM-GOX activity to the encapsulation efficiency. It was maximum in the presence of 0.5% chitosan, the next was 1.0% followed by 2% (Figure 7), which may have been induced by the low rate of passing through for the substrate and product of the enzyme because of the thicker coating and more compact lattice structure of the semi-permeable membrane. It also possibly blocked the binding site of load GOX with glucose, leading to a decrease in the apparent activity of loaded enzyme. Moreover, the aggregation of microcapsules in 2% chitosan affected the measurement of enzymatic activity, which explained the great deviation in enzymatic activity.

### 3.3. Thermal and Storage Stabilities of Free GOX and CACM-GOX

Free GOX solution containing 1 mg/mL enzyme protein in 0.2 M sodium phosphate buffer (pH 6.0) and CACM-GOX (dry weight of 33.65 mg/mL, pH 6.0) with an activity of 8.86 U/mL were kept at 4 °C and 25 °C, and residual activities of these samples were measured periodically for 60 days and 60 hours, respectively.

**Figure 8.**
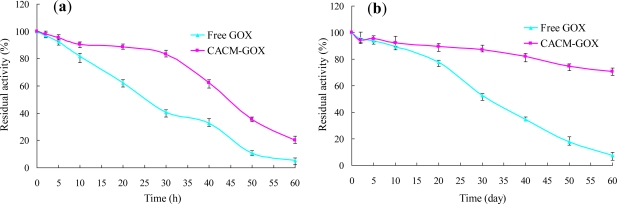
Thermal stabilities of free GOX and immobilized CACM-GOX (**a**) and storage stabilities of free GOX and immobilized CACM-GOX (**b**).

The immobilized GOX was much more thermally stable than the free. After 30 h incubation at 25 °C, free and immobilized GOX retained activities of 15.5% and 83.0%, respectively. Subsequently, the activity of CACM-GOX clearly decreased as seen in Figure 8a, merely 20.3% total activity was retained after 60 h at 25 °C. This phenomenon was also found by Onda, Ariga and Kunitake [28], the GOX film activity decreased drastically after four weeks storage. According to them, one possible cause was that the protein was decomposed by bacterial growth.

The storage stabilities of the CACM-GOX and free GOX were investigated at 4 °C and the results are shown in Figure 8b. The immobilized GOX was also much more stable than the free with 70.4% activity retained compared with merely 7.5% for free GOX after 60 days storage.

## 4. Conclusions

The necessary conditions for successful encapsulation of GOX into CACM microspheres using an emulsification-internal gelation-enzyme adsorption-coating by chitosan method with high residual activity had been established.

Total activity of CACM-GOX and encapsulation efficiency were dependent on the adsorption medium pH with much higher activity and encapsulation efficiency at pH values near the p*I* of GOX. The high porous structure of CaAlg gel beads made by carbon dioxide release during the course of CaCO_3_ dissolution contributed to the high absorption efficiency of protein. The molecular weight of chitosan was a very important factor for the whole experiment. Chitosans with low molecular weight, 20,000 (LMW) and 100,000 (MMWa), can enter the interior of CACM with less space resistance, resulting in low encapsulation efficiency. Enzyme absorption occured mainly within the first 20 min and then the speed became slower. The encapsulation efficiency and GOX load, coating for 60 min under the condition of 1% chitosan, were 91.1% and 4.15 (mg/g), respectively. Low GOX load was beneficial for CACM-GOX application in flour and could make the extremely low dosage of enzyme disperse in flour matrix homogenously in favor of the release and catalysis of GOX. The specific CACM-GOX activity was remarkably influenced by the concentration of chitisan and the maximum was achieved in the presence of 1% chitosan in the experiment.

## Figures and Tables

**Table 1.** Effects of chitosan (MMWb) concentration and encapsulation time on the encapsulation efficiency of GOX, the amount of GOX loaded and the total activity of the immobilized CACM-GOX.

**Chitosan Concentration (%, w/w)**		**The Encapsulation Efficiency of GOX (%)**^a^			**The Amount of GOX Loaded (mg/g)**^a^			**The Total Activity of CACM-GOX (U)**^a,b^		

		**0.5**	**1.0**	**2.0**	**0.5**	**1.0**	**2.0**	**0.5**	**1.0**	**2.0**
Encapsulation time (min)	**10**	46.7 ± 2.7	56.3 ± 3.5	55.5 ± 1.9	25.1 ± 0.6	26.9 ± 0.3	25.0 ± 0.2	3.51 ± 0.21	4.19 ± 0.12	3.40 ± 1.21
	**20**	55.3 ± 2.2	70.3 ± 2.4	70.1 ± 1.1	31.5 ± 0.4	32.0 ± 0.7	31.3 ± 0.3	4.20 ± 0.27	5.11 ± 0.31	4.76 ± 1.19
	**40**	64.2 ± 1.9	83.1 ± 3.1	84.5 ± 0.7	35.2 ± 0.5	37.7 ± 0.5	32.2 ± 0.5	4.76 ± 0.28	6.05 ± 0.23	4.82 ± 1.09
	**60**	76.1 ± 3.2	91.1 ± 1.2	90.6 ± 2.0	37.2 ±0.3	41.5 ± 0.4	31.4 ± 0.3	5.32 ± 0.12	6.56 ± 0.24	5.72 ± 0.87
	**80**	75.2 ± 2.4	92.5 ± 2.7	93.6 ± 1.9	37.4 ± 0.7	41.6 ± 0.5	31.5 ± 0.4	5.35 ± 0.22	6.35 ± 0.16	5.93 ± 1.07

a Values are presented as mean ±S.D., *n* = 3;

b The CACM-GOX was made by incubating 2 mL CaAlg gel beads (dry weight of 7.7 mg/mL) in 1 mL of 0.2 M pH 4 buffer solution containing 1 mg/mL GOX for definite time and then coated with chitosan solution.
